# Multimodality neuroimaging in vascular mild cognitive impairment: A narrative review of current evidence

**DOI:** 10.3389/fnagi.2023.1073039

**Published:** 2023-03-15

**Authors:** Qiuping Liu, Xuezhu Zhang

**Affiliations:** ^1^First Teaching Hospital of Tianjin University of Traditional Chinese Medicine, Tianjin, China; ^2^National Clinical Research Center for Chinese Medicine Acupuncture and Moxibustion, Tianjin, China; ^3^Tianjin University of Traditional Chinese Medicine, Tianjin, China

**Keywords:** vascular mild cognitive impairment, multimodal neuroimaging, resting-state functional magnetic resonance imaging, diffusion tensor imaging, arterial spin labeled perfusion imaging

## Abstract

The vascular mild cognitive impairment (VaMCI) is generally accepted as the premonition stage of vascular dementia (VaD). However, most studies are focused mainly on VaD as a diagnosis in patients, thus neglecting the VaMCI stage. VaMCI stage, though, is easily diagnosed by vascular injuries and represents a high-risk period for the future decline of patients’ cognitive functions. The existing studies in China and abroad have found that magnetic resonance imaging technology can provide imaging markers related to the occurrence and development of VaMCI, which is an important tool for detecting the changes in microstructure and function of VaMCI patients. Nevertheless, most of the existing studies evaluate the information of a single modal image. Due to the different imaging principles, the data provided by a single modal image are limited. In contrast, multi-modal magnetic resonance imaging research can provide multiple comprehensive data such as tissue anatomy and function. Here, a narrative review of published articles on multimodality neuroimaging in VaMCI diagnosis was conducted，and the utilization of certain neuroimaging bio-markers in clinical applications was narrated. These markers include evaluation of vascular dysfunction before tissue damages and quantification of the extent of network connectivity disruption. We further provide recommendations for early detection, progress, prompt treatment response of VaMCI, as well as optimization of the personalized treatment plan.

## 1. Introduction

Due to the process of ageing, the incidence rates of cerebral vascular diseases and neurodegenerative diseases like Alzheimer’s disease (AD) and dementia have drastically increased (GBD, 2016). By 2050, the total number of dementia patients is expected to reach 1.52 million people. Data show that 25% of them will be from the Chinese population (GBD, 2016). As the second most common dementia after AD, VaD has a great impact on life quality of patients and brings a heavy burden to the family and society (Plassman et al., 2007). Owing to its high prevalence and potential reversibility, VaD has attracted great attention (O'brien, 2006; Plassman et al., 2007). As the precursor stage of VaD (Wentzel et al., 2001), VaMCI’ s early prediction and intervention play an important role in delaying the transition to VaD (Ravaglia et al., 2006; Jak et al., 2009; Jongsiriyanyong and Limpawattana, 2018). Therefore, early diagnosis and risk factor reduction are clinical strategies to delay the disease progression. At present, there is no reliable method for early diagnosis and recognition of VaMCI. In recent years，the American Academy of Neurology and European Academy of Neurology recommends the use of neuroimaging for the evaluation of dementia patients, due to its ability to identify the pathological cause of dementia syndrome and unearth reliable imaging markers for the early diagnosis (Knopman et al., 2001; Filippi et al., 2012).

Here, we review different multimodal neuroimaging methods such as rs-fMRI, DTI, ASL perfusion imaging, as well as their synergy for the diagnosis of VaMCI patients. The correlation of different neuroimaging features with the cognitive function of these patients is further summarized to provide recommendations for the successful evaluation of dementia progression and prevention by advanced and quantitative neuroimaging technologies.

## 2. Certain vascular risk factors underlie the pathological mechanisms of VaMCI

**Figure 1:**
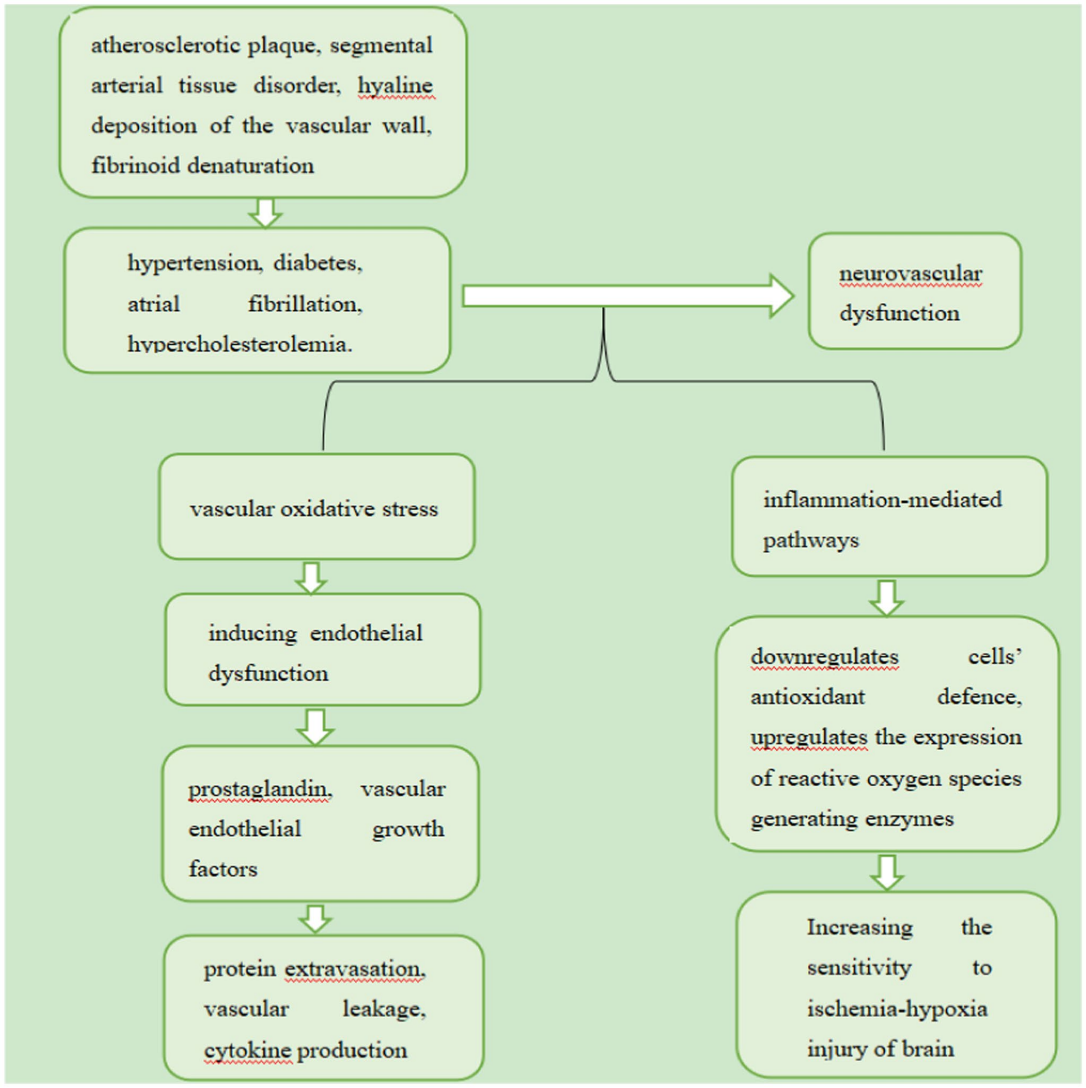
Certain vascular risk factors underlie the pathological mechanisms of VaMCI.

VaMCI is mainly induced by vascular risk factors, which include hypertension, diabetes, atrial fibrillation and hypercholesterolemia (Gorelick et al., 2011). They may induce neurovascular dysfunction through vascular oxidative stress and inflammation-mediated pathways. Oxidative stress promotes the release of prostaglandin and vascular endothelial growth factors by inducing endothelial dysfunction, which in turn promotes protein extravasation, vascular leakage, and cytokine production (Gorelick et al., 2011). On the other hand, inflammation downregulates cells’ antioxidant defence and upregulates the expression of reactive oxygen species generating enzymes (Gorelick et al., 2011; El-Sahar et al., 2021). This vicious cycle holds the potential to destroy the microenvironment of the brain, thus increasing its sensitivity to ischemia-hypoxia injury (Iadecola et al., 2009), Additionally, Vascular risk factors are related to various vascular pathologies, including atherosclerotic plaque, segmental arterial tissue disorder, hyaline deposition of the vascular wall and fibrinoid denaturation (Thal et al., 2012; Caplan, 2015). These vascular diseases reduce the cerebral blood flow (CBF) of perforating arteries, which supply subcortical nuclei, cortical projection fibres, and commissural fibres (Dey et al., 2016). As a result, connections between the cerebral cortex and subcortical regions, as well as between intracortical regions, are disrupted, which can lead to cognitive impairment (Dey et al., 2016). Certain vascular risk factors underlie the pathological mechanisms of VaMCI showed in the Figure 1.

## 3. Basic MRI features of VaMCI

Magnetic resonance imaging (MRI) is the key neuroimaging modality and has high sensitivity and specificity for detecting pathological changes, including small vessel disease (Mijajlović et al., 2017). A study indicated that the presence of moderate or severe white matter hyperintensities (WMH) on MRI is a hallmark of VaMCI, and extensive white matter damage in the temporal lobe, cingulate gyrus, bilateral lateral ventricles and other areas in VaMCI patients (Yu et al., 2017). Moreover, it was found that the number of lacunar infarcts in VaMCI was 3 times that in normal people, and white matter lesions, frontal Angle and third ventricle widening were also significantly more than in normal people (Meyer et al., 2005; Vermeer et al., 2007). Another studies revealed that VaMCI may display a single critical site infarction was sufficient to cause VaD (Snowdon et al., 1997), more than 2 multiple lacunar infarcts outside the brain stem (Snowdon et al., 1997; Chen et al., 2009), and Intracranial hemorrhage at critical sites, or ≥2 intracranial hemorrhage (Sachdev et al., 2014). Valenti and Li et al. reported that nearly one-third of VaMCI patients had at least one CMB and more than one-third had CMBs in multiple regions (Valenti et al., 2016; Li et al., 2021). They also found that more percentages of severe WMH, cerebral microbleeds (CMBs), enlarged perivascular spaces (EPVS) and cerebral atrophy compared with healthy controls.

## 4. Resting-state functional magnetic resonance imaging

### 4.1. Definition

rs-fMRI is a non-invasive neuroimaging technique that measures brain local functional connections at rest and is based on brain low-frequency (<0.1 Hz) MRI signal fluctuations with blood oxygen level dependence (BOLD; Biswal et al., 1995). Moreover, patients are scanned in quiet. It is generally accepted that rs-fMRI can effectively investigate brain networks. In other words, BOLD fMRI is applied to analyze the synchronization between individual cortical areas. Then, functional connections are delineated to illustrate the correlation between isolated regions and spontaneous neuron activities under resting-state (Biswal et al., 1995; Greicius et al., 2003; Fox and Raichle, 2007). Data show that these BOLD signals are not direct indicators of neuron activities. Instead, they reflect local fluctuation of deoxyhemoglobin concentration determined by blood flow, blood volume and oxygen metabolism (Raichle and Mintun, 2006). With advances in fMRI, the research on the pathways underlying the brain connections has expanded from the structural to the functional level of investigation. It has been revealed that the abnormal brain FC detected by the fMRI predated conventional structural changes (e.g., encephalotrophy) and clinical symptoms (Sheline and Raichle, 2013). Indeed, rs-fMRI provides a promising way to explore the changes of spontaneous neural activities related to various brain diseases (Greicius, 2008; Fox and Greicius, 2010). Changes in low-frequency BOLD signal fluctuations were observed in patients with AD, epilepsy and Parkinson’s disease (PD; He et al., 2007; Wu et al., 2009; Luo et al., 2011). Sun et al. reported abnormality of functional connections located between the posterior cingulate cortex (PCC) and frontal as well as temporal regions in patients with VaMCI (Sun et al., 2011). A study found that the functional activities of medial prefrontal cortex, bilateral cingulate gyrus/precuneus and left inferior parietal lobule in patients with AD or mild cognitive impairment (MCI) have changed (Zhang et al., 2012). Similarly, it has been reported that the decreased functional activity of five clusters including the right inferior temporal gyrus, the left medial prefrontal gyrus, the left anterior cingulate gyrus (ACG), the right wedge and the right middle occipital gyrus is related to the severity of AD (Wang et al., 2017).

### 4.2. Common clinical indicators of rs-fMRI

The ommon clinical indicators of rs-fMRI showed in Table 1.

**Table 1:** The common clinical indicators of rs-fMRI in VaMCI.

Indicators	ReHo	ALFF	FC	DC
Effects	Analyzing rs-fMRI signal local features Reflecting the local spontaneous neural activities Reflecting the abnormal changes of cognitive related brain function	Reflecting the size of spontaneous BOLD signals and examining spontaneous brain activities Reflecting local spontaneous neuron activities in the early phase of the disease fALFF effectively reduced the exhibited enhanced sensitivity and specificity of detected spontaneous neural activities due to interferences to physiological signals such as intracranial venous sinus and cerebrospinal fluids	Exploring connectivity patterns in specific brain regions Maping remote connections and detect hemodynamic responses in the brain detected by rs-fMRI not found by ReHo Reflecting abnormal patterns in specific brain regions	Focuses on the relationship between voxels and the entire network connection Objectively and comprehensively provide functional connectivity information of resting state in the whole brain network, which

#### 4.2.1. Regional homogeneity

ReHo refers to the correlation between a voxel time series and its locally adjacent voxel time series, which effectively quantifies the synchronization of the BOLD time series between a voxel and its locally adjacent voxel (Zang et al., 2004; Peng et al., 2016). It is an important method for analyzing rs-fMRI signal local features. As it reflects the local spontaneous neural activities it has been widely used to explore indigenous brain activities (Zang et al., 2004; Peng et al., 2016). Therefore, factors determining ReHo value include spatial adjacency and functional homogeneity of time series. These factors provide valuable spatiotemporal information from a neurobiological perspective (Jiang and Zuo, 2016). It has been further demonstrated that ReHo can be used as an imaging biomarker to monitor and/or identify AD pathology (Zhang et al., 2012). Additionally, ReHo has been demonstrated to be significantly associated with a patient’s cognitive performance (Zhang et al., 2012; Liu Y. et al., 2014). Data showed that alterations in intracranial atherosclerosis decrease CBF delivery and efficiency, resulting in the inconsistency of amplitude and/or phase of the BOLD signal of the single neural cluster. Therefore, it is generally accepted that a low ReHo value reflects impaired cerebral perfusion (Tu et al., 2020). Meanwhile, it was demonstrated that ReHo was successfully used in clinical research of various diseases, including attention deficit hyperactivity disorder (ADHD), AD and MCI (Zhang et al., 2012; Wang et al., 2013). Zuo et al. found that ReHo was significantly reduced in the left cerebellum and right lentiform nucleus of VaMCI patients (Zuo et al., 2019). Meanwhile, it was hypothesized that the low ReHo value might be related to diminished neuron activities as the mean ReHo value of the left ACG was negatively correlated with the trail making test (TMT; Tu et al., 2020). Additionally, ReHo reduction of VaMCI patients was closely related to MoCA scores, demonstrating that fMRI-based measurement might indicate brain dysfunction (Zuo et al., 2019). In patients with VaMCI, Diciotti et al. reported that a remarkably negative association between ReHo and MoCA scores, with higher ReHo in the left posterior cerebellum of patients with outstanding integral cognitive impairment, with higher ReHo in the middle cingulate cortex bilaterally of patients with worse executive functions. The findings revealed that ReHo is significantly correlated with measurements of the cognitive disorders (Diciotti et al., 2017). Orsolini et al. also revealed that patients with cognitive decline of cerebrovascular disease showed significantly lower ReHo in the right insula, the left superior frontal gyrus, and the bilateral anterior cingulated cortex, which belonging to networks involved in inhibition and attention (Orsolini et al., 2021). In short, the application of ReHo can reflect the abnormal changes of cognitive related brain function.

#### 4.2.2. Amplitude of low-frequency fluctuation

ALFF is a rs-fmri derived way that primarily measures the total power of the BOLD time course over a specific frequency range (0.01 ~ 0.08 Hz; Zang et al., 2007). It reflects the size of spontaneous BOLD signals and designs to examine spontaneous brain activities (Nugent et al., 2015). As a non-invasive technique of rs-fMRI, ALFF is more advanced than conventional MRI for the diagnosis of advanced pathological changes in cerebral vascular diseases and can reflect local spontaneous neuron activities in the early phase of the disease (Zang et al., 2007). Furthermore, the changes in local spontaneous neuron activities of ALFF can be detected in animals and humans (Logothetis et al., 2001; Moosmann et al., 2003; Pelled and Goelman, 2004; Yang et al., 2007). Recent studies indicated that cognitive impairment patients had abnormal ALFF within the PCC. For VaMCI patients, the alterations of ALFF in brain regions were predominantly found in the default mode network (DMN). Compared with the healthy control group, the ALFF was reduced in the bilateral medial prefrontal cortex (anterior DMN), precuneus (posterior DMN) and posterior parietal cortex (Yi et al., 2012; Wang et al., 2019; Zhuang et al., 2021). Moreover, the decrease in ALFF was positively correlated with the impairment of cognitive functions as assessed by Montreal Cognitive Assessment (MOCA), suggesting that the spontaneous neuron activities were associated with cognitive decline (Yang, 2021). Additionally, reduced ALFF in the precuneus was significantly correlated with the cognitive disability of AD and MCI patients measured by the minimum mental state examination (MMSE; Oakes et al., 2007; Wen et al., 2013).

Previous studies have revealed that ALFF is extensively affected by other physiological noises. Zou et al. proposed a method based on the fractional amplitude of low-frequency fluctuation (fALFF), which was defined as the ratio of ALFF and the given low-frequency band sum (Zou et al., 2008). Compared with ALFF, the fALFF effectively reduced the exhibited enhanced sensitivity and specificity of detected spontaneous neural activities due to interferences to physiological signals such as intracranial venous sinus and cerebrospinal fluids (Zou et al., 2008). For instance, fALFF has been widely applied in the diagnosis of AD (Yang et al., 2018), MCI (Pan et al., 2017), and the amnestic mild cognitive impairment (Zhou et al., 2020). Additionally, a study found that the fALFF values of right frontal lobe, left hippocampus and right cingulate gyrus were significantly increased in patients with cognitive impairment after acute cerebellar infarction, and the fALFF value of posterior cerebellum decreased significantly (Fan et al., 2019).

#### 4.2.3. Functional connections

Seed-based FC analysis is a correlation analysis method for exploring connectivity patterns in specific brain regions (Sala-Llonch et al., 2015). Anatomically separated brain regions are found to fluctuate synchronously and exhibit strong FC, thus forming a complex functional network (Biswal et al., 1995). FCs between different brain regions correspond well to their nerve fiber connections, indicating a strong anatomical basis for FCs (Greicius et al., 2009; Honey et al., 2009). In addition, FC has also been proved to be closely related to regional CBF and metabolism, such as regions with strong connections show more obvious CBF (Liang et al., 2013), higher oxygen consumption (Wu et al., 2009) and glucose metabolism (Tomasi et al., 2013). Therefore, it can be used to map remote connections and detect hemodynamic responses in the brain detected by rs-fMRI not found by ReHo (Peng et al., 2016). Multiple literatures have shown that FC is also associated with dynamic changes in local neuronal ensemble activity, which reflects the neural flexibility or the dynamic range that affects the adaptability and efficiency of the nervous system (Garrett et al., 2013; Nomi et al., 2017). Regional neural variability and brain network dysfunction (Dennis and Thompson, 2014; Colasanti et al., 2016) in patients with stroke (Kielar et al., 2016), multiple sclerosis (AS; Petracca et al., 2017), AD (Scarapicchia et al., 2018) and other neurological disorders (Zoller et al., 2017). Some researches suggesting that FC is highly relevant to the cognitive performance of a specific field, including DMN, executive control network and dorsal attention network, all of which are closely related to attention and execution (Barkhof et al., 2014; Shaw et al., 2015). In the FC analysis, the selections for regions of interest (ROIs) are not consistent. Existing studies show that the main focus is mainly on the PCC connections and their critical role in brain cognitive function and memory (Ding et al., 2015). Ding et al. reported that FC of PCC and the left thalamus were significantly reduced in patients with VaMCI (Ding et al., 2015). In VaMCI patients, a significant reduction of FC was found in the right inferior frontal gyrus, the right middle frontal gyrus, bilateral precentral gyrus, and the right postcentral/superior parietal lobule (Zuo et al., 2019). Additionally, MOCA scores were positively correlated with the decrease of FC in the anterior cingulate cortex and posterior parietal cortex, suggesting that the local FC was related to cognitive impairment (Wang et al., 2021). In brief, FC can reflect abnormal patterns in specific brain regions.

#### 4.2.4. Degree centrality

DC is a graph-based brain network measurement method, which calculates the temporal correlation between a single voxel and other intracerebral voxels within a mask at the voxel level (Zuo et al., 2012). In other words, Consider each voxel as a node and calculate the number of functional connections between each node and other nodes (Buckner et al., 2009). The larger the DC of a node, the more important the node is in the whole brain network, and the stronger its information communication abilities (Zhu et al., 2019). DC focuses on the relationship between voxels and the entire network connection, also be used to detect abnormal changes in functional connectivity in brain (Buckner et al., 2009; Gao et al., 2016). Unlike ALFF which reflects local brain activities, voxel-level DC can objectively and comprehensively provide functional connectivity information of resting state in the whole brain network, which is different from traditional functional neural research methods such as regional homogeneity (Zuo et al., 2012; Adriana et al., 2013). It explains the relationship betweesn the local brain activities and the whole brain network. Moreover, compared with other methods such as ALFF and ReHo (Huang et al., 2015; Shao et al., 2015), DC does not involve defining ROIs and assessing connectivity across the human brain at voxel level (Zuo and Xing, 2014; Shao et al., 2015), which can provide valuable information for the changes of nodes in human brain connections caused by diseases (Adriana et al., 2013). It has high repeatability (Zuo and Xing, 2014). At present, DC has been widely used to explore the neurobiological mechanism and pathophysiological mechanism of brain network changes in various diseases (Lou et al., 2015; Shen et al., 2015). Therefore, DC method has attracted a lot of attention and has been used to explore the neural mechanisms of several diseases, such as Alzheimer’s disease (Guo et al., 2016), alcohol dependence (Luo et al., 2017), attention deficit hyperactivity disorder (Wang et al., 2017), and Parkinson’s disease (Guo et al., 2020). Abnormal DC has been observed in MCI, AD and PD patients (Grau-Olivares et al., 2010; Li et al., 2017). Studies have found that increased DC values in temporal gyrus and hippocampus may be associated with impaired memory function (Feng et al., 2021). Yang et al. demonstrated that DC reduction was significantly correlated with the Hamilton Anxiety Scale (HAMA) in VaMCI patients, suggesting that VaMCI patients may be more likely to develop symptoms of anxiety (Yang, 2021). Existing literature reported that many studies combined DC and FC to explore changes in functional patterns in patients with neurological diseases (Cui et al., 2016; Guo et al., 2020).

## 5. Diffusion tensor imaging

The hyperintensity in White Matter (WM) is associated with impaired executive and overall cognitive function of the brain (Dao et al., 2018). Specifically, the white matter tract is essential for the maintenance of the normal brain cortex and cortico-subcortical connections. Therefore, the integrity of WM plays a key role in the synchronous activities and neural activation of the brain functional network. DTI can be used as a sensitive method to explore the neural mechanisms of different cognitive impairments (Liu et al., 2019). It is an MRI technique that directly measures the integrity of the brain white matter (WM; Wang et al., 2013). Compared with conventional techniques, it is more sensitive in detecting cognitive impairments and has a higher correlation with the patient’s cognitive function, especially in the early stage of neurological diseases (Xu et al., 2010; Wang et al., 2013). It explores the integrity of WM in patients with dementia or cognitive impairments, and even detects minor changes in the complex brain structural networks, creating the great potential to discover early stages of the disease and to optimize personalized treatment regimens (Sabri et al., 1999; Buckner et al., 2009; Xu et al., 2010; Wang et al., 2013). Recent studies revealed that the assessment for correlation between the WM damage and the cognitive function by DTI was superior to those by T2 weighted or FLAIR sequences (O’Sullivan et al., 2004a,b). In other words, the dispersion of lesions and normal white matter on DTI was increased, and the average diffusion rate of normal white matter was related to the performance of functional tests. These correlations remain significant after controlling age, gender, brain volume and T1/T2 lesion volume (O’Sullivan et al., 2004a,b). No significant correlation between neuropsychological scores of T2 lesions. Additionally, DTI appeared as the most sensitive technique to assess structural WM microstructural damage in patients with cerebrovascular diseases (Banerjee et al., 2016). It was further demonstrated that DTI reflected the processes of the white matter tracts (e.g., cortical tract and spinal tract) and the overall extent and shape of the water proton diffusion by measuring the water proton diffusivity inside the brain tissue (Nir et al., 2013). It also finally clarifies the microstructural integrity of WM. It was demonstrated that fractional anisotropy (FA) appeared as a widely used DTI measurement method in clinical studies to describe diffusion anisotropy. It was further demonstrated that DTI was very sensitive to changes in the integrity of WM and neuron connections, with higher values indicating stronger axonal integrity and lower values suggesting incomplete or loss of neuron connections (Nir et al., 2013).

Another common method of DTI measurement is the mean diffusivity (MD), which is used to characterize the pattern of water diffusion within a tissue, reflecting the average amplitude of diffusion in various directions. Research found that MD was seen to correlate negatively and FA to correlate positively with global and selective cognitive performance in patients with VaMCI (Xu et al., 2010). Moreover, FA and MD correlated with patients’ memory and attention executive scores (Xu et al., 2010). Additionally, FA and MD within the cingulate bundle correlated with verbal memory scores in non-demented elderly with cerebral small vessel disease, while MD in the frontal lobe was associated with psychomotor speed performance (Tuladhar et al., 2015). Mascalchi et al. reported that patients of VaMCI with increased MD substantially corresponded to area of decreased FA in WM exhibiting. The result reveals that a substantially symmetric damage of the long WM tracts in terms of increased MD and decreased FA emerged (Mascalchi et al., 2014). Another study found that MoCA scores were positively correlated with FA as well as MD (negative correlation) of almost the global cerebral hemispheres to the patients with VaMCI, in an almost symmetrical fashion. The study indicates that the cognitive deficits are consistently sustained by the microstructural damage of the normal-appearing WM revealed by DTI (Mascalchi et al., 2019). In summary, loss of microstructural integrity in WM is usually reflected in decreased FA and/or increased MD (Beaulieu, 2002; Sen and Basser, 2005).

## 6. Arterial spin labelling

ASL perfusion imaging is a promising non-invasive tool for assessing CBF, which can be used to discover certain vascular features of early cognitive impairment (Yoshiura et al., 2009; Iturria-Medina et al., 2016). It is generally accepted that imaging of CBF patterns not only provides direct information of cerebral tissue perfusion but has also been used as a marker for the functional integrity of brain tissue (Roman and Pascual, 2012). It has been widely used to clinically evaluate patients with cognitive impairments (Roman and Pascual, 2012). Data show that CBF changes may be present even in asymptomatic dementia risk individuals (Thambisetty et al., 2010; Lunau et al., 2012; Okonkwo et al., 2014). This indicates that CBF measurements hold the ability to detect subclinical brain pathologies. Additionally, the reduction in total CBF was not only confirmed to be present in dementia patients but was also associated with structural signs of brain ageing and cognitive decline in non-dementia individuals (Bisschops et al., 2004; Ruitenberg et al., 2005; Appelman et al., 2008; Vernooij et al., 2008). In summary, cerebral hypoperfusion may appear at an early stage of cognitive function impairment. Meanwhile, other authors’ results showed that alterations in CBF also reflected the possible effects of vascular risk factors (Pase et al., 2012; Henriksen et al., 2014) especially those that led to encephalopathy (Meltzer et al., 2000; Zonneveld et al., 2015).

Numerous evidence indicated that cerebral hypoperfusion caused by vascular diseases led to neuron and astrocyte death, impaired brain volume and neuron function, thereby serving as a biomarker for cognitive decline (Jack et al., 2010; Wierenga et al., 2014). Henriksen et al. revealed that typical CBF changes preceded the appearance of subjective cognitive difficulties (Henriksen et al., 2017). Global CBF was shown to be associated with encephalatrophy, ischemic lesions and cognitive decline (Ruitenberg et al., 2005; Appelman et al., 2008; Vernooij et al., 2008). Moreover, it was shown that the ischemic brain injury and cognitive changes suggested a decrease of global CBF (Brundel et al., 2012; van der Veen et al., 2015; Zonneveld et al., 2015). It was demonstrated that the cerebral flow correlated with cognition in patients with VaD, in whom the CBF was significantly reduced mainly in the frontal, parietal, and temporal cortices (Schuff et al., 2009; Gao et al., 2013).

## 7. Synergy of multimodality neuroimaging

### 7.1. Synergy of BOLD and ASL

As is one of the most important factors contributing to VaMCI (Pasi et al., 2015), the cerebral small vessel disease (CSVD) may cause endothelial cell damage, abnormal perfusion, and disruption of the brain structure as well as damage in brain functional connections (Wallin et al., 2018; Thrippleton et al., 2019), which in turn results in dysregulation of the neurovascular unit (NVU) composed of neurons, astrocytes, and blood vessels (Muoio et al., 2014). The NVU plays a crucial role in maintaining the homeostasis and the normal function of the brain microenvironment (Pasley and Freeman, 2008; Helman and Murphy, 2016). Under physiological conditions, the microvascular flow matches well with neurons and astrocytes in the NVU, termed neurovascular coupling (Girouard, 2006). The occurrence of CSVD may disturb its coupling and lead to disorders in cerebral blood supply and neuron activities, which are regarded as the main possible cause of cognitive impairment (Caruso et al., 2019; Moretti and Caruso, 2020). Currently, most studies focused mainly on ASL perfusion imaging to find CBF perfusion or on a single imaging technique that embodied neuronal activities, which did not comprehensively reflect dysregulated neurovascular coupling. Many authors suggest that CBF fusion and neuron activities be regarded as a functional complex (Liu et al., 2021). Other authors’ results showed that FC in brain regions had a similar pattern to CBF, which generated synergy with cerebral perfusion and neuronal activities in different voxels (Liang et al., 2013; Zhu et al., 2013; Jann et al., 2015). The significant correlation between BOLD and ASL perfusion imaging, respectively, represented by the two is a measurable indicator. The abnormal CBF distribution was found to be consistent with FC changes in VaD patients (Schuff et al., 2009; Gao et al., 2013). Liu et al. combined ReHo with CBF *via* the synergy of BOLD and ASL perfusion imaging and used the overall ReHo-CBF correlation coefficient and ReHo/CBF ratio to measure the intrinsic links between neuron activities and vascular responses (Liu et al., 2021). They found that the overall ReHo-CBF correlation was significantly lower, and the ReHo/CBF ratio was significantly abnormal in patients with cognitive impairment compared with healthy individuals. It showed an indication of more severe neurovascular coupling impairment in the patients with cognitive impairment. Meanwhile, the study suggested that a coupling exists between cerebral perfusion and functional activities in patients with mild cognitive impairment. It indicated abnormal neurovascular coupling in the early stages, and the development of the disease might be related to disease severity and cognitive impairment (Liu et al., 2021).

In summary, data show that the ReHo-CBF correlation coefficient measured the spatial distribution consistency between cerebral blood supply and neuron activities at the whole brain voxel level, while ReHo/CBF ratio represented the strength of connections between the neuron supplied by a CBF unit and the surrounding brain areas (Liu et al., 2021).

### 7.2. Interactions between the structure and function of cerebrum

The human brain has been modelled as a large-scale integrated complex network in functional and structural domains (Zhang et al., 2011). It is increasingly recognized that the anatomical constraints are imposed on FC by structural connections in the network (Honey et al., 2009). In turn, FC exerts influence on the structural connections through the brain plasticity (Hagmann et al., 2010; Guerra-Carrillo et al., 2014). The demonstration of the association between structure and function holds huge potential to expand our understanding of how the association between brain structure and function affects human cognition and behavior (Wang et al., 2015). This close and complex association is of increasing interest to researchers and will contribute to a better understanding of the intrinsic integration of neural resources and will advance our knowledge about the neuropathological basis of brain - related diseases. Here, we present an conclusion of the existing literature on the imaging approaches for examination of brain pathologies in patients to explore the relationship between the functional abnormalities in the brain and its structural damage which further targeted elucidation of the related central mechanisms underlying neurological pathologies.

#### 7.2.1. Synergy of BOLD-fMRI and DTI

Both DTI and BOLD-fMRI as discussed previously are highly sensitive modalities for measuring the structural and functional brain abnormalities in cognitively impaired patients. The close association between the brain structure and function indicates the integration of these two modalities as a valuable tool for exploring the pathogenesis of brain pathologies (Ye and Bai, 2018). DTI and BOLD imaging of spontaneous neuron activities showed the relationship between the brain structure connections (Koch et al., 2002; De Luca et al., 2006). Thus proving them as tools for identifying subtle WM changes and intrinsic connections between different cortical areas (Hagmann et al., 2008; van Eimeren et al., 2010). DTI and fMRI have been increasingly shown to detect early structural and functional brain alterations related to VaMCI, especially in the last 2 years (Ye and Bai, 2018). A close link between them has been confirmed by a series of studies (van Eimeren et al., 2010; Khalsa et al., 2014). The researches also revealed that brain regions showing prominent FC are also structurally connected in DTI anatomy, regions presenting stronger FC are also more significantly connected in structure (Hagmann et al., 2008; Khalsa et al., 2014). Moreover, it has been documented that the structures with abnormal DTI were generally consistent across the brain regions associated with abnormal FC changes (Ye and Bai, 2018). Therefore, Combining DTI and fMRI findings may be highly valuable for the study of early and specific brain alterations in VaMCI.

#### 7.2.2. Synergy of BOL-fMRI and VBM

Voxel-based morphometry (VBM) obtains anatomical information of a patient’s brain by high-resolution 3D T1 weighted imaging and utilizes the voxel-based cortical morphometric analysis method to quantitatively calculate the cortical grey matter (GM) density or volume of each 3D voxel in the brain structure images. Then the differences of anatomical structure were obtained, and the changes of gray matter in brain were evaluated. As a common measure in VBM measurements, the brain grey matter volume (GMV) predicts the presence of cognitive impairment and the rate of cognitive decline (Mungas et al., 2002). In addition to cerebral alterations directly related to cerebrovascular damage, the global or local GM atrophy may also be responsible for cognitive impairment in patients with cerebral vascular diseases (Laakso et al., 1996; Fein et al., 2000; Mungas et al., 2001). A VBM study showed extensive volume atrophy in patients with VaMCI, especially in the frontal cortex and subcortical regions (Thong et al., 2014). Specifically, in the study of Seo, the thinning of frontal cortex in patients VaMCI is closely related to executive dysfunction (Seo et al., 2010). Fein et al. also found that the severity of cognitive impairment is significantly correlated with GM atrophy, and the GM atrophy of hippocampus and frontal lobe are predictors of brain cognitive impairment (Fein et al., 2000; Mungas et al., 2001, 2002; Mok et al., 2005). Moreover, the GM atrophy was shown to reflect the loss of neurons or other types of neuropathological outcomes (Markus, 2007). Consequently, GM atrophy has been a cause of cognitive decline (e.g., memory loss, attention/executive dysfunction, language impairment, visuospatial function, and depression of VaMCI patients; Lei et al., 2016; Li et al., 2017; Lyu et al., 2019).

Because the fact that regions of interest were not predefined, VBM provided unbiased whole-brain comparisons between the studied patients’ groups, making it an ideal method for exploratory cross-sectional studies (Stebbins et al., 2016). Hence, the established synergy between BOL-fMRI and VBM provided comprehensive visualization of characteristic changes in brain function and structure. As an early stage of VD, VaMCI is regarded as the most important subtype of vascular disease caused by CSVD (Peng, 2019). Indeed, CSVD was typically accompanied by changes in the brain function and structure (Ter Telgte et al., 2018) and GMV atrophy was observed in the frontal, temporal versus parietal cortical areas, pons, thalamus, caudate nucleus, and hippocampus of VaMCI patients (Seo et al., 2010, 2012; Thong et al., 2014). These morphological changes interacted with functional activities (Koch et al., 2002; Liu C. et al., 2014; Tu et al., 2020). For patients with ischemic vascular diseases, GM atrophy strongly correlated with dementia severity and it was an independent predictor for the cognitive decline of patients with cerebral vascular diseases (Fein et al., 2000; Mungas et al., 2002). Yang et al. found that GMV of the right precentral gyrus and right inferior temporal gyrus of VaMCI patients decreased (Yang, 2021) and this was related to ALFF reduction, indicating that CSVD patients might be exposed to impairments of the brain structure and function (Zhao et al., 2015; Lyu et al., 2019).

## 8. Technical problems or difficulties

Although many neuroimaging studies have reported the brain structure and functional characteristics related to cognitive function in patients with VaMCI, most of the previous studies evaluated the single-modality image information. Due to the different imaging principles, the data provided by single-modality images are limited. In contrast, multi-modality magnetic resonance imaging can provide multiple comprehensive data such as tissue anatomy function and find imaging markers related to the occurrence and development of VaMCI, but there are still some technical problems to be solved: (1) The b value selected in each study is different, which needs to be optimized combined with signal-to-noise ratio, image quality and scanning time. (2) Different research institutions use different scanning instruments, imaging parameters and reconstruction algorithms, which can improve the comparability between different studies by establishing standardized image acquisition and post-processing processes. (3) The method of manually drawing the region of interest is greatly affected by subjective factors, and semi-automatic or automatic segmentation techniques can be used to improve the repeatability of imaging parameters. (4) By establishing standardized data analysis methods and evaluation methods, the comparison and feasibility of different studies are improved. Different data analysis methods and evaluation methods may affect the analysis of structure and function.

## 9. Conclusion

This article conducts a narrative review in the clinical applications of multimodal MRI diagnostic approaches for VaMCI. The close association between the brain structure and function indicates that the integration of rs-fMRI, DTI and ASL patterns may be valuable for the detailed neuroimaging exploration of the pathogenesis of VaMCI. These findings provide a valuable basis for a better understanding of VaMCI pathophysiological mechanisms, its unique features for recognition and diagnosis, as well as provide suggestions for possible targets in the development and evaluation of new cognitive and pharmacological interventions of VaCI pathologies. In future, we can conquer the limits of single-modality analysis and advance the diagnostic and prognostic potential on single patient examination.

## Author contributions

QL contributed to the conceptualization of the study, literature search. XZ and QL performed the quality appraisal and manuscript development. All authors contributed to the article and approved the submitted version.

## Funding

This work has received funding by National Natural Science Foundation of China (No. 82174492) and Tianjin Science and Technology Plan Project (No. 20YFZCSY00810).

## Conflict of interest

The authors declare that the research was conducted in the absence of any commercial or financial relationships that could be construed as a potential conflict of interest.

## Publisher’s note

All claims expressed in this article are solely those of the authors and do not necessarily represent those of their affiliated organizations, or those of the publisher, the editors and the reviewers. Any product that may be evaluated in this article, or claim that may be made by its manufacturer, is not guaranteed or endorsed by the publisher.
